# Protein intrinsic disorder and influenza virulence: the 1918 H1N1 and H5N1 viruses

**DOI:** 10.1186/1743-422X-6-69

**Published:** 2009-06-03

**Authors:** Gerard Kian-Meng Goh, A Keith Dunker, Vladimir N Uversky

**Affiliations:** 1 Center for Computational Biology and Bioinformatics, Indiana University School of Medicine, Indianapolis, Indiana 46202, USA; 2 Institute for Intrinsically Disordered Protein Research, Indiana University School of Medicine, Indianapolis, Indiana 46202, USA; 3 Institute for Biological Instrumentation, Russian Academy of Sciences, 142290 Pushchino, Moscow Region, Russia; 4 Institute of Molecular and Cell Biology, Singapore 138673, Republic of Singapore

## Abstract

**Background:**

The 1918 H1N1 virus was a highly virulent strain that killed 20–50 million people. The cause of its virulence remains poorly understood.

**Methods:**

Intrinsic disorder predictor PONDR^® ^VLXT was used to compare various influenza subtypes and strains. Three-dimensional models using data from X-ray crystallographic studies annotated with disorder prediction were used to characterize the proteins.

**Results:**

The protein of interest is hemagglutin (HA), which is a surface glycoprotein that plays a vital role in viral entry. Distinct differences between HA proteins of the virulent and non-virulent strains are seen, especially in the region near residues 68–79 of the HA_2_. This region represents the tip of the stalk that is in contact with the receptor chain, HA_1_, and therefore likely to provide the greatest effect on the motions of the exposed portion of HA. Comparison of this region between virulent strains (1918 H1N1 and H5N1) and less virulent ones (H3N2 and 1930 H1N1) reveals that predicted disorder can be seen at this region among the more virulent strains and subtypes but is remarkably absent among the distinctly less virulent ones.

**Conclusion:**

The motions created by disorder at crucial regions are likely to impair recognition by immunological molecules and increase the virulence of both the H5N1 and the 1918 H1N1 viruses. The results help explain many puzzling features of the H5N1 and the 1918 H1N1 viruses. Summarizing, HA (and especially its intrinsically disordered regions) can serve as a predictor of the influenza A virulence, even though there may be other proteins that contribute to or exacerbate the virulence.

## Background

### Introducing influenza viruses

Influenza viruses belong to the *Orthomyxoviridae *family of negative sense, single-stranded, segmented RNA viruses. This family contains five genera, classified by variations in nucleoprotein (NP and M) antigens: influenza A, influenza B, influenza C, thogotovirus, and isavirus [1]. Influenza A viruses, which are a major cause of influenza in humans, have multiple subtypes that are labeled according to an H number (for hemagglutinin) and an N number (for neuraminidase). There are 16 different hemagglutinin (HA) antigens (H1 to H16) and nine different neuraminidase (NA) antigens (N1 to N9) for influenza A [2]. The influenza A viral genome consists of 8 genes encoding 11 proteins, including: two nonstructural proteins (NS1 and NS2), four transcriptase proteins (PB2, PB1, PB1-F2, and PA), two surface glycoproteins (HA and NA), two matrix proteins (M1 and M2), and one nucleocapsid protein (NP), where the 3 extra proteins arise from the use of different reading frames.

The influenza A virion (i.e. a complete virus particle with its RNA core and protein coat) is a globular particle sheathed in a lipid bilayer derived from the plasma membrane of its host. Two integral membrane proteins, HA and NA, are studded in the lipid bilayer. They are distributed evenly over the virion surface, forming characteristic spike-shaped structures. The matrix formed by matrix proteins M1 and M2 is located beneath the envelope. This matrix encompasses eight pieces of genomic RNA, each in association with many copies of a nucleoprotein (NP), some "non-structural" proteins with various functions (e.g. NS1 and NS2) and several molecules of the three subunits of its RNA polymerase. The influenza pandemics (i.e. the epidemics of the influenza virus) of the recent era include the Spanish influenza (H1N1, 1918), the Asian influenza (H2N2, 1958), and the Hong Kong influenza (H3N2, 1968) [3-6]. The human tolls resulting from the pandemics have been very large. The Spanish influenza pandemic of 1918–1919 alone caused acute illness in 25–30% of the world's population and resulted in the death of 40 million people worldwide. The number of the deaths in the US have been estimated at 500,000, 70,000 and 34,000, respectively, for the Spanish influenza, the Asian influenza, and the Hong Kong influenza pandemics [3,4,6] (for more information see also ).

More recently, various subtypes have afflicted smaller numbers of humans mainly via avian species, especially poultry. The subtypes include H5N1, H7N3, H7N7 and H9N2. For instance, in 2003, an outbreak of H7N7 occurred in the Netherlands, where 89 people were confirmed to have H7N7 influenza virus infection following an outbreak in poultry on several farms. One death was recorded, whereas most of the 89 patients suffered only conjunctivitis and mild influenza symptoms. As for the related H7N3, which resulted in quarantine of 18 North American farms in 2004 to halt the spread of the virus, only two human cases were reported, but none resulted in fatalities [7]. H5N1 subtypes, on the other hand, generally have higher fatality rates of above 50% [3]. For example, in 1997, the avian H5N1 virus was transmitted from poultry to humans in Hong Kong and caused 18 human cases of influenza with a high mortality rate [8]. In February 2003, two cases of H5N1 infection in humans occurred in Hong Kong, one of them fatal [9]. In 2004–2005, during an extensive outbreak of H5N1 infection in poultry in eight countries of Southeast Asia, over 100 human cases occurred in Vietnam, Thailand and Cambodia, 50% of them fatal [10]. A few human cases of H9N2 have been reported [11]. It has been reported that H9N2 bears striking genetic resemblance to H5N1 [12].

In summary, different strains of influenza have led to widely divergent fatality rates. Our goal here is to find sequence features that correlate with, and therefore might help to explain, this divergence.

### Hemagglutinin

Specific surface glycoproteins are used by the enveloped viruses, such as influenza, human immunodeficiency virus-1 (HIV-1), and Ebola, to enter target cells via fusion of the viral membrane with the target cellular membrane [13-15]. One of the most well-studied membrane fusion proteins is the influenza virus HA, which is a homotrimeric type I transmembrane surface glycoprotein responsible for virus binding to the host receptor, internalization of the virus, and subsequent membrane-fusion events within the endosomal pathway in the infected cell. HA is also the most abundant antigen on the viral surface and harbors the primary neutralizing epitopes for antibodies. Each 70-kDa HA subunit contains two disulfide-linked polypeptide chains, HA_1 _and HA_2_, created by proteolytic cleavage of the precursor protein HA_0 _[16]. Such a cleavage is absolutely crucial for membrane fusion [16]. During membrane fusion, HA binds the virus to sialic acid receptors on the host cell surface and, following endocytosis the acidic pH (pH 5–6) of endosomal compartments, induces dramatic and irreversible reorganization of the HA structure [17].

The HA trimer has a tightly intertwined "stem" domain at its membrane-proximal base, which is composed of HA_1 _residues 11 to 51 and 276 to 329, and HA_2 _residues 1 to 176. The dominant feature of this stalk region in the HA trimer is the three long, parallel α-helices (~50 amino acids in length each), one from each monomer, that associate to form a triple-stranded coiled coil. The membrane-distal domain consists of a globular "head", which is formed by HA_1 _and which can be further subdivided into the R region (residues 108–261), containing the receptor-binding site and major epitopes for neutralizing antibodies, and the E region (residues 56–108 and 262–274), with close structural homology to the esterase domain of influenza C HA esterase fusion (HEF) protein [18]. The HA_2 _chain contains two membrane-interacting hydrophobic peptide sequences: an N-terminal "fusion peptide" (residues 1–23), which interacts with the target membrane bilayer [19], and a C-terminal transmembrane segment that passes through the viral membrane.

Crystallographic studies suggested that the interaction with the host cell involves a dramatic structural reorganization of HA_2_, which moves the fusion peptide from the interior approximately 100 Å toward the target membrane [20,21]. In this process, the middle of the original long α-helix unfolds to form a reverse turn, jack-knifing the C-terminal half of the long α-helix backward toward the N-terminus. These molecular rearrangements place the N-terminal fusion peptide and the C-terminal transmembrane (TM) anchor at the same end of the rod-shaped HA_2 _molecule [22,23], facilitating membrane fusion by bringing the viral and cellular membranes together.

### Causes of influenza virulence remain largely elusive

The understanding of the causes of virulence for the respective subtypes of influenza viruses remains largely incomplete. This is clearly illustrated by the example of the H1N1 virus, for which several hypotheses have been put forth to explain its virulence. One theory proposes that the H1N1 virulence could be attributed to the effective inhibition of type I interferon by the NS1 protein from the 1918 H1N1 virus. However, this model is unable to account for the virulence of H5N1 subtypes that have dissimilar NS1 sequences [24]. Multiple gene mutations in various viral proteins (HA, NA and polymerases) have also been suggested as a requirement for high virulence, especially in mice [3]. Adding to the confusion is the finding that viruses with 1918-H1N1 HA are highly pathogenic to mice [3,25]. The reason for this remains elusive. We hope that by using a relatively new set of computational tools (e.g. protein disorder prediction), we can provide suggested answers to some of these questions, suggestions to be followed up by additional experiments.

### Other puzzles of influenza

There is yet another mystery related to influenza and connected to the mysterious way in which the 1918 H1N1 virus suddenly disappeared by 1920, after raging relentlessly in 1918 and 1919 [5,6,26]. Several strains of H1N1 appeared but none of them was as virulent as the 1918 virus [24,27,28]. How did these re-appearing strains differ from the 1918 H1N1 virus? How did they evolve? Is the H1N1 and the H5N1 virulence coming from the same protein, e.g. HA? We report here that disorder prediction can shed at least some light on these mysteries too.

### Protein intrinsic disorder

Many proteins are intrinsically disordered; i.e. they exist as dynamic ensembles of interconverting structures instead of possessing rigid 3-D structures under physiological conditions *in vitro*. Intrinsically disordered proteins [29] are known by several names including "intrinsically unstructured" [30] and "natively unfolded" [31-33]. These proteins carry out numerous biological functions that are unparalleled by ordered proteins [29,30,32,34-51]. Importantly, it has been recently shown that the availability and abundance of intrinsically disordered proteins inside a cell is under tight control [52,53]. Intrinsically disordered proteins and regions differ from structured globular proteins and domains with regard to many attributes, including amino acid composition, sequence complexity, hydrophobicity, charge, flexibility [29,32,54], and type and rate of amino acid substitutions over evolutionary time [55]. Many of these differences between ordered and intrinsically disordered proteins were utilized to develop various disorder predictors [56-60]. The disorder predictors used in this work are two different Predictors of Naturally Disordered Regions, PONDR^®^s VLXT and VL3 [61-64]. Earlier, we have shown that different HA subtypes possess detectable differences in predicted disorder [65]. Here we continue this investigation to examine if the patterns of predicted disorder in the HAs of the various subtypes show any correlation with virulence.

## Results

### Summary of percentage predicted disorder of various HA subtypes

Influenza A subtypes are traditionally identified by their HA and NA proteins. Table 1 provides an overview of the percentage of predicted disorder found in HA_1 _and HA_2 _chains of various influenza A subtypes whose crystal structures were resolved. Table 1 shows that H1N1 and H5N1 subtypes have several variants, such as 1918 H1N1 and 1930 H1N1.

**Table 1:** Summary Table of Percentage of Predicted Disorder in Each Chain of Influenza Hemagglutinins.

Viral Subtype	Accessions	Strain	Description	%VLXT		
				HA1	HA2	(HA1)^b^
H1N1	1ruz	A/South Carolina/1/18	Spanish Flu 1918	12	12	
	1ruy	A/Swine//Iowa/15/30	Swine 1930	15	5	Ordered
	1rvx	A/Puerto Rico/3/34	1934	11	6	

H3N2	1mqn	A/Duck/Ukraine/63	Hong Kong Flu 1968	25 19		More Disordered

H5N1	2fk0	A/Vietnam/1203/2004	Avian Flu	15	18	Ordered
	2ibx	A/Vietnam/1119/2004		12	12	Ordered
	1jsn	A/Duck/Singapore/3/97		15	19	Ordered

H9N2	1jsd	A/Swine/HongKong/9/98	Swine 1998	15	20	Ordered

The samples used are those elucidated using X-ray crystallography.

^a^Percentage of residues that are predicted to be disordered by PONDR^@^VLXT in a given chain

^b^Qualitative description based on predicted disorder for HA_1 _Chain

### Three-dimensional presentation of predicted disorder in various HA subtypes

**Figure 1:**
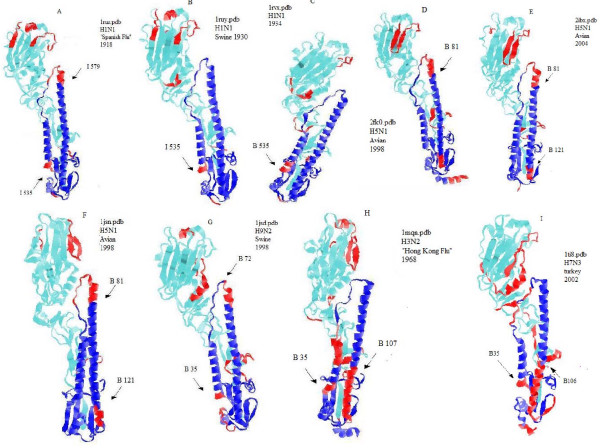
**Three-dimensional representation of the HA with predicted disorder annotation**. The regions in red represent areas predicted to be disordered by VLXT. Conversely, the blue areas represent regions predicted to be ordered. A) 1918 H1N1 HA_2_, 1ruz.pdb B)1930 H1N1 1ruy.pdb, C) 1934 H1N1, 1rvx.pdb D) H5N1, 2fk0.pdb E) H5N1, 2ibx.pdb F) H5N1, 1jsn.pdb G) H9N2.pdb, Swine H) H3N2, "Hong Kong Flu-1968 " Progenitor, Sample from 1963 I) H7N3, Avian.

In order to detect differences in the distribution of predicted intrinsic disorder in the various HA subtypes, a series of three-dimensional models from proteins characterized by X-ray crystallography were generated. Figure 1 shows a set of models generated by the combination of X-ray data and results of the PONDR^® ^VLXT analysis. The HA1 and HA2 subunits are shown in light turquoise and dark blue colors. The areas predicted to be disordered by PONDR^® ^VLXT are marked by red color. Figure 1 clearly shows that various HA subtypes differ dramatically in the amount and localization of the predicted intrinsic disorder.

### PONDR^® ^VLXT plots with normalized B-factor scores

**Figure 2:**
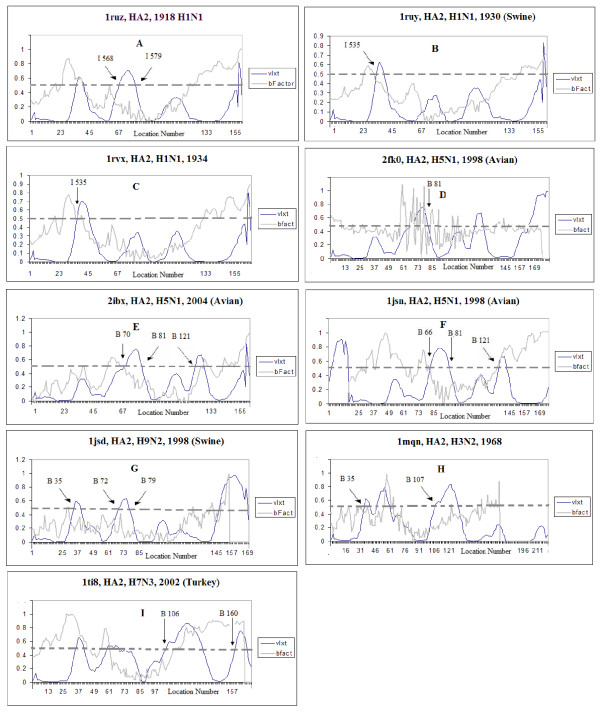
**PONDR^@ ^VLXT and B-factor plots of the HA_2_**. The blue curves represent the VLXT score, while the black ones represent the normalized B-factor. A) 1918 H1N1 HA2, 1ruz.pdb B)1930 H1N1 1ruy.pdb, C) 1934 H1N1, 1rvx.pdb D) H5N1, 2fk0.pdb E) H5N1, 2ibx.pdb F) H5N1, 1jsn.pdb G) H9N2, 1jsd.pdb, Swine H) H3N2, "Hong Kong Flu-1968 " Progenitor, Sample from 1963, 1mqn.pdb I) H7N3, Avian, H7N3, Italy, Avian, 1it8 [24,22,20].

Figure 2 compares the distributions of predicted disorder within the sequences of HA_2 _subunits from several influenza A subtypes and strains with the distributions of normalized B-factor scores evaluated from X-ray structures of the corresponding subunits. A residue with a PONDR^® ^VLXT score at or above 0.5 is considered to be disordered. For convenience, some areas predicted to be disordered are marked by arrows and corresponding labels pointing to the residues. Figure 2 shows that there is a general correlation between the high level of predicted disorder in a given HA region and its high B-factor scores. This indicates that HA regions of predicted disorder can gain some fixed structure during crystallization, although these regions possess high mobility even in the crystal structures.

**Figure 3:**
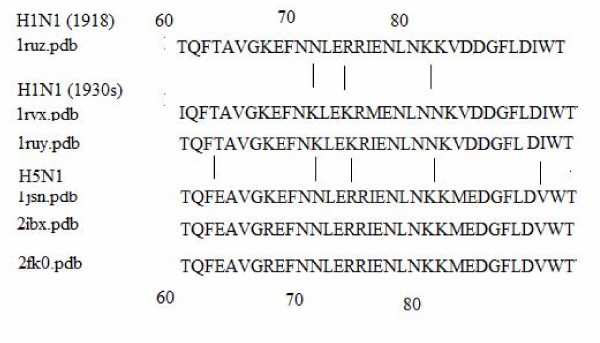
**Protein sequence of HA_2 _around tesidue 68**. Vertical lines show where mutations have taken place. The numbers denote the residue numbers.

Figure 3 shows the peculiarities of the amino acid sequences of HA_2 _subunits from the various influenza subtypes and strains in the vicinity of the residue 70. As will be seen from the subsequent discussion, this region might be related to the virulence of influenza viruses.

## Discussion

### H1N1 1918 viral strain has predicted disorder at a crucial region of HA

#### A specific region is predicted to be disordered in the 1918 H1N1 but not in other H1N1 strains

As mentioned in the introduction, the 1918 pandemic caused global devastation in 1918–19, but became nonexistent by 1920 [24,27,28,66]. However, the H1N1 subtype of influenza A was still present in 1934. To answer the question of why the 1918 strain was highly virulent, we compared the intrinsic disorder distributions in amino acid sequences of HA proteins from the 1918 strain and later strains. Analysis of the data in Table 1 shows that the overall percentage of predicted disorder does not vary much for the different strains of H1N1, and that all of the HA proteins could be considered as ordered by prediction. However, a comparison of three-dimensional models enhanced by the PONDR^® ^VLXT annotations revealed marked differences between various HA subtypes. The HA_2 _protein of the H1N1 1918 strain has two distinct regions of predicted disorder (starting at positions 35 and 68, respectively, see Figures 1A and 2A), whereas two other strains from the 1930s reveal only one disordered region (starting at position 35, see Figures 1B–C and 2B–C). The region that was predicted to be disordered in the 1918 strain but not in the H1N1 strains after 1919 is a region at or around position number 68 of the HA_2 _protein. The high level of predicted disorder in the region around position 35 seems to be a common feature among strains of the subtype H1N1. This region lies at the base of the HA stalk. The missing region of predicted disorder among the H1N1 strains in the 1930s, around position 68, is located at the higher end (the "tip") of the stalk. A superficial analysis of the HA structure (Figure 1) suggests that this region is likely to be located in an area close the HA center of gravity, suggesting that motions of HA_2 _at this region are likely to have greatest impact on the HA_1 _subunits, where the exposed area of the protein lies.

### The crucial region of predicted disorder is associated with virulent strains

#### A specific region of predicted disorder observed in 1918 H1N1 is seen in both H5N1 and H9N2

A noticeable feature of the HA_2 _subunit in the H5N1 subtype is the presence of two specific regions of predicted disorder. The first region lies again at the base of the stalk, even though on a different α-helix (see Figure 1). The other region of predicted disorder coincides with the disordered fragment seen in the 1918 virus; i.e. this region is positioned at the tip of the HA_2 _stalk (around residue 68) and is quite similar in size to that in the 1918 strain. This region is also seen in the H9N2 subtype (See Figures 1E and 3E), but is noticeably shorter (residues 72–79).

#### The crucial region of predicted disorder in 1918 H1N1 is replaced by more ordered regions in many less virulent strains

Given the data we have discussed thus far, one might suggest that the region around position 68 could be commonly predicted to be disordered among all influenza subtypes. However, the H7N3 subtype shown in Figures 1G and 2G does not have any predicted disorder near position 68. This is also the case for the H3N2 subtype shown in Figures 1F and 2F. Interestingly, the Hong Kong H3N2 virus and the avian H7N3 virus, both of which have ordered helical tips of HA_2_, were less virulent than the Spanish influenza strain where this tip is predicted to be disordered.

#### The low pathogenic avian influenza H7N3 strain does not have predicted disorder at the helical tip

Infection of humans by avian influenza H7 subtypes has been described in the Introduction. The human symptoms of the H7N3 have been largely mild, suggesting that in the majority of cases we are dealing with low pathogenic avian influenza (LPAI) strains. Although some highly pathogenic avian influenza (HPAI) strains do exist, the particular sample available via PDB (1ti8.pdb, see Figures 1I and 2I); i.e. A/Turkey/Italy/02/, is a representative of the LPAI strains [67,68] (Table 1). A visual inspection of the three-dimensional structure (Figure 1I) reveals some interesting characteristics. The crucial region of predicted disorder observed in the highly virulent 1918 H1N1 strain (residues 68–79) is absent in this H7 subtype. An inspection of regions in close proximity to the helical tip (HA_2 _residues 68–79) reveals that the adjacent loop (residues 61–68, see Figure 2I) is predicted to be disordered. This suggests that H7-related viruses could become more pathogenic with mutations that extend the disordered region to include the helical tip.

### The H9N2 puzzle and modulation of virulence

#### A modulating "switch" in H9N2: infectivity versus immune evasion

The H9N2 virus is generally non-virulent for mammals [69]. However, our analysis revealed that it has a region of predicted disorder at the tip of its helical stalk. This region of predicted disorder in the H9N2 HA_2 _is noticeably shorter than that in the 1918 H1N1 protein. On the other hand, studies on H9N2 strains with low virulence revealed that a single point mutation can make these viruses more virulent [69]. In fact, Leu226 in the HA receptor-binding site (RBS), responsible for human virus-like receptor specificity, was found to be important for transmission of the H9N2 viruses in ferrets [69]. Interestingly, earlier it has been shown that changes in the HA receptor-binding site of H9N2 isolates allow adaptation to mammalian-type sialic receptors [70] and that a change at position 227 of the HA_1 _subunit of H5N1 had a noticeable effect on its virulence in mice [71]. This suggests a mechanism by which the virus can potentially modulate its virulence. The virulence arising from mutations near the receptor binding site may work in tandem with the immune evasion arising from tip of the HA_2 _stalk. One way that the former can modulate the effects of the latter is by controlling the infectivity of the virus. A virus that binds to its host with great efficiency and is also effective in evading the immune systems creates the "perfect storm" for virulence.

#### A secondary switch for virulence of H9N2 and H5N1via oligosaccharides

It has been suggested that immune pressure caused by the use of a vaccine could create a survival advantage for those influenza viruses that undergo antigenic variation [72]. This evolution produces low-virulence escape mutants, many of which were associated with the acquisition of new glycosylation sites in the HA_1 _subunit, at positions 198 and 131 in H9N2 and H5N1, respectively [73,74]. It has been reported that the restored pathogenicity of low-virulence H5 and H9 escape mutants by lung-to-lung passage in mice could be attributed not only to reacquisition of the wild-type HA gene sequence, but was also associated either with the removal of a glycosylation site (the one acquired previously by the escape mutant) without the exact restoration of the initial wild-type amino acid sequence, or, for an H5 escape mutant that had no newly acquired glycosylation sites, with an additional amino acid change in a remote part of the HA molecule (at position 156 of HA_1_) [75]. This clearly shows the crucial role of the loss of glycosylation sites in restoration of the virulence of H9 and H5 readaptants. It should be noted that mutations affecting the glycosylation of HA are likely to affect virulence, since it has been proposed that it is common for the glyco-conjugate to act in tandem with intrinsic disorder of viral proteins in immune evasion [76,77]. Therefore, it should not be surprising that modulation of the virulence will often involve changes at the glyco-conjugate.

#### Possible mechanism for initial wave of non-virulent strain in 1918

By analogy, this may also help explain why and how the first wave of the 1918 H1N1 virus was not as virulent as the second [24,28]. As little as a single mutation could have set off the virulent potential of the second wave of the 1918 H1N1 virus.

### Immune evasion, virulence, and the 'cytokine storm'

#### Avian influenza viruses and antigenic shift

It is believed that one of the major reasons for the inability of the global population to have effective neutralizing antibodies against the viruses (including the influenza virus A) is the predisposition of the viral genomes for the genetic reassortment, which results from the segmented structure of genomes. As a result of this genome segmentation, shuffling of gene segments can occur if two different subtypes of influenza A virus infect the same host cell [78]. This is an important mechanism, as all combinations of the 16 different HA antigens (H1 to H16) and 9 different NA antigens (N1 to N9) are found in water fowl, whereas only H1 to 3 and N1 to 2 viral subtypes are commonly found in humans with influenza. Therefore, if a human H3N2 virus and an avian H5N1 virus co-infect a human, the genetic reassortment can produce a novel H5N2 virus, which then can be efficiently transmitted from human to human because the majority of the gene segments apart from H5 come from the human virus. This shuffling of gene segments obviously leads to significant antigenic changes, known as antigenic shift, as a result of which most of the population would not have any effective neutralizing antibodies against the new virus subtype [78]. In turn, this lack of effective antibodies can be responsible for high virulence of new virus subtype.

#### Crucial disordered regions associated with avian influenza viruses

A consistent trend that has been observed is the tendency for the crucial disordered HA_2 _region around positions 67–79 to be seen among avian or avian-related viruses (Figures 1A, D–G, I). In fact, all H5N1 and H9N2 samples that we analyzed have this predicted disordered region (although it is shortened in the H9N2 (Figure 1G)). Even the avian-related H7 subtype (Figures 1I, 2I) has a tinge of disordered at the edge of this region. This suggests that avian-related influenza viruses depend on this region for their fitness. They possibly utilize disorder in this region to evade the immune systems of various hosts that they try to move into.

#### Crucial disordered regions not commonly associated with human influenza viruses

By contrast, except for the 1918 H1N1 strain, most human influenza A viruses have not been observed to have this disordered region at the HA_2 _(Figures 1B, C, H and 2B, C, H). This is especially the case for viruses that have been circulating in humans for long period of time. A hint for such pattern can be seen in the evolution of the H1N1 virus after 1918 [5,6]. The virulent 1918 H1N1 virus became extinct by 1920 [18]. Subsequent H1N1 viruses were not as virulent as the 1918 H1N1 strain [6]. This correlates with our inability to find any H1N1 samples from dates after 1918 with the "fangs"(i.e. crucial disordered region around positions 67–79, Figure 1B, C), even though some of the samples from the 1930s were probably descendents of the 1918 virus given the similarity of the predicted disorder patterns of their HA_1 _(Figure 1A–C).

#### "Cytokine storm" and intrinsic disorder in HA

Most subtypes of avian influenza virus, such as H9, cause very mild disease in poultry. However, the H5 and H7 subtypes are known to cause outbreaks involving massive deaths in domestic poultry [78]. What is even worse avian influenza A viruses can be zoonotically transmitted to humans leading to serious outbreaks of human sickness caused by avian virus. Such outbreaks can be very deadly. For example, the 1997 outbreak of H5N1 influenza in Hong Kong Special Administrative Region (HKSAR) involved 18 human cases, with six fatalities [79,80].

The fatality rate of avian influenza epidemic (>50%) occurred in Southeast Asia in 1997 was significantly higher compared to the pandemic caused by the 1918 H1N1 (5–10%). When considering the fatal/total case numbers (208/340) reported by World Health Organization in respect of December 14th, 2007, the mortality rate has reached to 61 percent [81].

One of the reasons for the high mortality rates associated with certain subtypes of avian influenza were attributed to so-called "cytokine storm" or hypercytokinemia, which is characterized by the extremely enhanced production and secretion of large numbers and excessive levels of pro-inflammatory cytokines [81]. This hypercytokinemia is a result of the overactive inflammatory response related to the virus-induced cytokine dysregulation. In fact, H5N1 viruses were shown to serve as very strong inducers of various cytokines and chemokines, such as TNF-α, IFN-γ, IFN-α/β, IL-1, IL-6, IL-8, MIP-1, MIG, IP-10, MCP-1, and RANTES, leading to the "cytokine storm". This "cytokine storm" is believed to be responsible for the development of lethal clinical symptoms such as extensive pulmonary oedema, acute bronchopneumoniae, alveolar haemorrhage, reactive haemophagocytosis, and acute respiratory distress syndrome, associated with necrosis and tissue destruction [81]. It has been reported than mutations in NS1, PB2, HA and NA could be responsible for the initiation of the cytokine storm. These mutations can increase the viral replication rate, expend the tissue tropism, facilitate the systemic invasion and increase the resistance of a virus against the host antiviral response [81]. We believe that the crucial disordered region around positions 67–79 discussed in our paper can contribute to the virus resistance toward the host immune system and therefore can be one of the factors provoking the "cytokine storm" in humans affected by avian H5N1 (or related) influenza.

### Sequential analysis: shuffling and grouping by residues of the same polarity around position 68 of HA_2_

#### Shuffling by polarity in virulent strains

An analysis of the HA_2 _sequence of the 1918 H1N1 strain shows that a set of polar residues located in the vicinity of residue 68 make this segment more polar and, thus, potentially more disordered. Figure 2 shows that the normalized B-factor values vary with PONDR^® ^VLXT values. However, the effects seem to be reduced in many cases. For example, it can be seen that a peak in the normalized B-factor curve near position 60 in the 1918 H1N1 HA_2 _plot (Figure 2A, 1ruz.pdb), corresponds to the PONDR^® ^VLXT maximum in the vicinity of residues 68–79. This crucial PONDR^® ^VLXT maximum, seen in the 1918 H1N1 and H5N1 HA_2_, is also seen on a smaller scale in the 1930s H1N1 HA_2_. The peak of the 1918 H1N1 HA_2 _is relatively higher than those from the 1930s, suggesting that this region in the 1918 H1N1 HA_2 _is more flexible. This is further supported by the corresponding B-factor curves (see Figure 2).

### Swine versus avian influenza viruses: patterns of predicted disorder

#### Predicted disorder patterns at the base of the stalk seem to be dependent on the hosts

Another puzzle is the presence of predicted disorder at the bottom of the stalk. Often the predicted disorder appears at the bottom of the longer alpha helix, but sometimes it appears at the base of the smaller helix. An example of the former is the H1N1 subtype, whereas an example of the latter is H5N1. In yet other cases, such as H7N3 and H3N2, predicted disorder appears at the bases of both helices. A hint for a possible explanation of this behavior is provided by the analysis of the swine variant of the H9N2 subtype (see Figures 1G and 2G) [82]. In this instance, a region of predicted disorder appears at the bottom of the longer α-helix, just as in H1N1. This is of particular interest since H1N1 is believed to be of swine-host origin [28,82]. Therefore, we suggest that the position of this predicted disorder region at the stalk bottom is dependent on the host type. Interestingly, the 1918 H1N1 is suspected to be of both avian and swine origins [24,83-85] just as the swine strain of the H9N2 subtype is of avian origin [82,86]. The pattern of predicted disorder of the 1918 H1N1 HA, which resembles that of H9N2, may therefore cast more light on the origin of the 1918 H1N1 virus. The theory that the 1918 H1N1 virus is of avian origin but evolved in swine [24,83] seems to be supported here.

## Conclusion

### Virulence of influenza A and intrinsic disorder

#### Virulence tied to the predicted disorder at segment at to the tip of the α-helix stalk

It can be seen that all the virulent strains analysed in this study have a region of predicted disorder at the top of the largest alpha helix of the HA stalk (near the residues 68–79, see Figures 1 and 2). This is the case for the 1918 H1N1 strain and for all the virulent samples of the H5N1 and H9N2 subtypes. Although the similarity between H9N2 and H5N1 is striking, the pathogenesis of H9N2 is still relatively unknown since few human cases have been reported so far. Our data suggest that the H9 subtypes are likely to be highly virulent as their HA_2 _contains the predicted disorder region similar to segment of predicted disorder seen in all of the virulent strains studied. Conversely, the subtypes and strains that are known to be less virulent than the 1918 H1N1 and H5N1 have no predicted disorder at the tip of the stalk. Examples of this behavior are H3N2 and the particular strain of H7N3.

#### H9N2 and 1918 H1N1: masking the virulence

The H9N2 subtype is potentially able to modulate its virulent nature by affecting the efficiency of binding to the host cells, thus controlling its ability to evade the immune system. This is supported by the observation that a single mutation near the receptor binding site is generally sufficient to convert H9N2 and some H5N1 strains from non-virulent to highly pathogenic. This also represents a potential mechanism by which the second wave of the 1918 H1N1 virus became more virulent than the first wave.

#### Virulence, evasion of immune response and intrinsic disorder

We propose that the high mobility of the exposed region of the HA trimer accounts for the evasion of the initial immune response. This highly dynamic nature of the potentially immunogenic region weakens the binding of antibodies and other immune response-related molecules to the HA molecule. In this way, the virus buys some time to invade the host. It is also possible that the immune system does not elicit the adequate immune response throughout all stages of the disease as tight binding to the highly dynamic HA is difficult. Evidence supporting both hypotheses can be found in the observation that when the 1918 H1N1 virus was introduced to mice, unusually high viral loads were seen within a short period of time [25]. The crucial region of predicted disorder at the helical tip is seen in all HAs of influenza viruses of avian origin analysed thus far. It is highly plausible that this disordered region is needed for the virus to move between various species of birds by allowing sufficient copies of the virion to avoid immune detection in the host's body in order to increase the odds of infections. Therefore, the increased intrinsic disorder may be associated with increased fitness of the virus.

#### Strategies for vaccine development against the pathogenic viruses

Development of vaccine for highly virulent viruses such as 1918 H1N1 or H5N1 may be fraught with dangers as a result of the ability of their proteins to trigger fatal immune responses [87]. The results here provide new potential strategies to develop vaccines for virulent influenza strains. Relatively non-virulent immunogenic proteins could be developed in either of two ways. One way is to use disorder predictors such as PONDR^® ^VLXT to quickly identify variants of the virulent viruses that are predicted to be non-virulent by lacking the region of predicted disorder at the tip of the stalk. Another strategy would be to mutate the crucial region in the viruses themselves at the crucial sequence in the HA_2 _protein.

## Methods

### Implementation details

The list of viral proteins of interest incorporates the proteins of Orthomyxoviruses. Searches were done on the list using the Entrez website. Available samples were randomly chosen with preferences given to those with longer chains and those with binding partners. Whenever possible, homologous proteins from different virus strains were included as samples and annotated. The respective FASTA and PDB [88] files were downloaded and stored using a JAVA^® ^program. The database design has been described in a previous paper [77].

### PONDR^® ^VLXT and B-factor normalization plots

The B-Factor values were retrieved from the respective PDB files and placed in the respective table in the MYSQL database already designed and populated [77]. The PONDR^® ^VLXT and normalized B-factor graphs were plotted using MS-EXCEL^® ^via output files obtained from SQL. The normalized B-factor values were calculated using EXCEL spreadsheet.

### Graphics tools

Three-dimensional graphics was developed utilizing Jmol [89] in conjunction with JAVA programming. The JAVA-JDBC program reads from the prediction information stored in the MYSQL^® ^database and generates the corresponding Jmol script, which creates the necessary molecular graphics for the protein.

## Competing interests

The authors declare that they have no competing interests.

## Authors' contributions

GKMG proposed the idea of the study, implemented the experiments, carried out the analyses, and drafted the manuscript. AKD helped to design experiments and participated in the manuscript drafting. VNU coordinated the studies, participated in their design and helped to draft the manuscript. All authors read and approved the final manuscript.

## References

[B1] Brooks GF, Butel JS, Morse SA (2004). Jawetz, Melnick and Adelberg's medical microbiology.

[B2] Fouchier RA, Munster V, Wallensten A, Bestebroer TM, Herfst S, Smith D, Rimmelzwaan GF, Olsen B, Osterhaus AD (2005). Characterization of a novel influenza A virus hemagglutinin subtype (H16) obtained from black-headed gulls. J Virol.

[B3] Garcia-Sastre A, Whitley RJ (2006). Lessons learned from reconstructing the 1918 influenza pandemic. J Infect Dis.

[B4] Reid AH, Taubenberger JK, Fanning TG (2004). Evidence of an absence: the genetic origins of the 1918 pandemic influenza virus. Nat Rev Microbiol.

[B5] Reid AH, Taubenberger JK, Fanning TG (2001). The 1918 Spanish influenza: integrating history and biology. Microbes Infect.

[B6] Kilbourne ED (2006). Influenza pandemics of the 20th century. Emerg Infect Dis.

[B7] Tweed SA, Skowronski DM, David ST, Larder A, Petric M, Lees W, Li Y, Katz J, Krajden M, Tellier R (2004). Human illness from avian influenza H7N3, British Columbia. Emerg Infect Dis.

[B8] (1998). Update: isolation of avian influenza A(H5N1) viruses from humans – Hong Kong, 1997–1998. MMWR Morb Mortal Wkly Rep.

[B9] Wuethrich B (2003). Infectious disease. An avian flu jumps to people. Science.

[B10] World Health Organization (2005). Avian influenza, Viet Nam – update. Wkly Epidemiol Rec.

[B11] Guan Y, Shortridge KF, Krauss S, Webster RG (1999). Molecular characterization of H9N2 influenza viruses: were they the donors of the "internal" genes of H5N1 viruses in Hong Kong?. Proc Natl Acad Sci USA.

[B12] Lin YP, Shaw M, Gregory V, Cameron K, Lim W, Klimov A, Subbarao K, Guan Y, Krauss S, Shortridge K (2000). Avian-to-human transmission of H9N2 subtype influenza A viruses: relationship between H9N2 and H5N1 human isolates. Proc Natl Acad Sci USA.

[B13] Skehel JJ, Wiley DC (1998). Coiled coils in both intracellular vesicle and viral membrane fusion. Cell.

[B14] Skehel JJ, Wiley DC (2000). Receptor binding and membrane fusion in virus entry: the influenza hemagglutinin. Annu Rev Biochem.

[B15] Eckert DM, Kim PS (2001). Mechanisms of viral membrane fusion and its inhibition. Annu Rev Biochem.

[B16] Wiley DC, Skehel JJ (1987). The structure and function of the hemagglutinin membrane glycoprotein of influenza virus. Annu Rev Biochem.

[B17] Skehel JJ, Bayley PM, Brown EB, Martin SR, Waterfield MD, White JM, Wilson IA, Wiley DC (1982). Changes in the conformation of influenza virus hemagglutinin at the pH optimum of virus-mediated membrane fusion. Proc Natl Acad Sci USA.

[B18] Stevens J, Corper AL, Basler CF, Taubenberger JK, Palese P, Wilson IA (2004). Structure of the uncleaved human H1 hemagglutinin from the extinct 1918 influenza virus. Science.

[B19] Durrer P, Galli C, Hoenke S, Corti C, Gluck R, Vorherr T, Brunner J (1996). H+-induced membrane insertion of influenza virus hemagglutinin involves the HA2 amino-terminal fusion peptide but not the coiled coil region. J Biol Chem.

[B20] Wilson IA, Skehel JJ, Wiley DC (1981). Structure of the haemagglutinin membrane glycoprotein of influenza virus at 3 A resolution. Nature.

[B21] Bullough PA, Hughson FM, Skehel JJ, Wiley DC (1994). Structure of influenza haemagglutinin at the pH of membrane fusion. Nature.

[B22] Weber T, Paesold G, Galli C, Mischler R, Semenza G, Brunner J (1994). Evidence for H(+)-induced insertion of influenza hemagglutinin HA2 N-terminal segment into viral membrane. J Biol Chem.

[B23] Wharton SA, Calder LJ, Ruigrok RW, Skehel JJ, Steinhauer DA, Wiley DC (1995). Electron microscopy of antibody complexes of influenza virus haemagglutinin in the fusion pH conformation. Embo J.

[B24] Taubenberger JK, Reid AH, Janczewsk TA, Fanning TG (2001). Integrating historical, clinical and molecular genetic data in order to explain the origin and virulence of the 1918 Spanish influenza virus. Philos Trans R Soc Lond B Biol Sci.

[B25] Kobasa D, Takada A, Shinya K, Hatta M, Halfmann P, Theriault S, Suzuki H, Nishimura H, Mitamura K, Sugaya N (2004). Enhanced virulence of influenza A viruses with the haemagglutinin of the 1918 pandemic virus. Nature.

[B26] Reid A (2005). The effects of the 1918–1919 influenza pandemic on infant and child health in Derbyshire. Med Hist.

[B27] Kilbourne ED (2006). Influenza pandemics of the 20th century. Emerg Infect Dis.

[B28] Reid AH, Taubenberger JK, Fanning TG (2001). The 1918 Spanish influenza: Integrating history and biology. Microbes Infect.

[B29] Dunker AK, Lawson JD, Brown CJ, Williams RM, Romero P, Oh JS, Oldfield CJ, Campen AM, Ratliff CM, Hipps KW (2001). Intrinsically disordered protein. J Mol Graph Model.

[B30] Wright PE, Dyson HJ (1999). Intrinsically unstructured proteins: re-assessing the protein structure-function paradigm. J Mol Biol.

[B31] Weinreb PH, Zhen W, Poon AW, Conway KA, Lansbury PT (1996). NACP, a protein implicated in Alzheimer's disease and learning, is natively unfolded. Biochemistry.

[B32] Uversky VN, Gillespie JR, Fink AL (2000). Why are "natively unfolded" proteins unstructured under physiologic conditions?. Proteins.

[B33] Uversky VN, Gillespie JR, Millett IS, Khodyakova AV, Vasiliev AM, Chernovskaya TV, Vasilenko RN, Kozlovskaya GD, Dolgikh DA, Fink AL (1999). Natively unfolded human prothymosin alpha adopts partially folded collapsed conformation at acidic pH. Biochemistry.

[B34] Dunker AK, Garner E, Guilliot S, Romero P, Albrecht K, Hart J, Obradovic Z, Kissinger C, Villafranca JE (1998). Protein disorder and the evolution of molecular recognition: theory, predictions and observations. Pac Symp Biocomput.

[B35] Dunker AK, Brown CJ, Lawson JD, Iakoucheva LM, Obradovic Z (2002). Intrinsic disorder and protein function. Biochemistry.

[B36] Uversky VN, Oldfield CJ, Dunker AK (2005). Showing your ID: intrinsic disorder as an ID for recognition, regulation and cell signaling. J Mol Recognit.

[B37] Daughdrill GW, Pielak GJ, Uversky VN, Cortese MS, Dunker AK, Buchner J, Kiefhaber T (2005). Natively disordered proteins. Protein Folding Handbook.

[B38] Dunker AK, Cortese MS, Romero P, Iakoucheva LM, Uversky VN (2005). Flexible nets. The roles of intrinsic disorder in protein interaction networks. FEBS J.

[B39] Xie H, Vucetic S, Iakoucheva LM, Oldfield CJ, Dunker AK, Obradovic Z, Uversky VN (2007). Functional anthology of intrinsic disorder. 3. Ligands, post-translational modifications, and diseases associated with intrinsically disordered proteins. J Proteome Res.

[B40] Vucetic S, Xie H, Iakoucheva LM, Oldfield CJ, Dunker AK, Obradovic Z, Uversky VN (2007). Functional anthology of intrinsic disorder. 2. Cellular components, domains, technical terms, developmental processes, and coding sequence diversities correlated with long disordered regions. J Proteome Res.

[B41] Xie H, Vucetic S, Iakoucheva LM, Oldfield CJ, Dunker AK, Uversky VN, Obradovic Z (2007). Functional anthology of intrinsic disorder. 1. Biological processes and functions of proteins with long disordered regions. J Proteome Res.

[B42] Uversky VN (2002). What does it mean to be natively unfolded?. Eur J Biochem.

[B43] Uversky VN (2002). Natively unfolded proteins: a point where biology waits for physics. Protein Sci.

[B44] Uversky VN (2003). Protein folding revisited. A polypeptide chain at the folding-misfolding-nonfolding cross-roads: which way to go?. Cell Mol Life Sci.

[B45] Tompa P (2002). Intrinsically unstructured proteins. Trends Biochem Sci.

[B46] Dyson HJ, Wright PE (2005). Intrinsically unstructured proteins and their functions. Nat Rev Mol Cell Biol.

[B47] Tompa P (2005). The interplay between structure and function in intrinsically unstructured proteins. FEBS Lett.

[B48] Galea CA, Wang Y, Sivakolundu SG, Kriwacki RW (2008). Regulation of cell division by intrinsically unstructured proteins: intrinsic flexibility, modularity, and signaling conduits. Biochemistry.

[B49] Dunker AK, Oldfield CJ, Meng J, Romero P, Yang JY, Chen JW, Vacic V, Obradovic Z, Uversky VN (2008). The unfoldomics decade: an update on intrinsically disordered proteins. BMC Genomics.

[B50] Dunker AK, Silman I, Uversky VN, Sussman JL (2008). Function and structure of inherently disordered proteins. Curr Opin Struct Biol.

[B51] Oldfield CJ, Meng J, Yang JY, Yang MQ, Uversky VN, Dunker AK (2008). Flexible nets: disorder and induced fit in the associations of p53 and 14-3-3 with their partners. BMC Genomics.

[B52] Gsponer J, Futschik ME, Teichmann SA, Babu MM (2008). Tight regulation of unstructured proteins: from transcript synthesis to protein degradation. Science.

[B53] Uversky VN, Dunker AK (2008). Biochemistry. Controlled chaos. Science.

[B54] Campen A, Williams RM, Brown CJ, Meng J, Uversky VN, Dunker AK (2008). TOP-IDP-scale: a new amino acid scale measuring propensity for intrinsic disorder. Protein Pept Lett.

[B55] Brown CJ, Takayama S, Campen AM, Vise P, Marshall TW, Oldfield CJ, Williams CJ, Dunker AK (2002). Evolutionary rate heterogeneity in proteins with long disordered regions. J Mol Evol.

[B56] Ferron F, Longhi S, Canard B, Karlin D (2006). A practical overview of protein disorder prediction methods. Proteins.

[B57] Radivojac P, Iakoucheva LM, Oldfield CJ, Obradovic Z, Uversky VN, Dunker AK (2007). Intrinsic disorder and functional proteomics. Biophys J.

[B58] Uversky VN, Radivojac P, Iakoucheva LM, Obradovic Z, Dunker AK (2007). Prediction of intrinsic disorder and its use in functional proteomics. Methods Mol Biol.

[B59] Dosztanyi Z, Sandor M, Tompa P, Simon I (2007). Prediction of protein disorder at the domain level. Curr Protein Pept Sci.

[B60] Dosztanyi Z, Tompa P (2008). Prediction of protein disorder. Methods Mol Biol.

[B61] Li X, Romero P, Rani M, Dunker AK, Obradovic Z (1999). Predicting protein disorder for N-, C-, and internal regions. Genome Inform Ser Workshop Genome Inform.

[B62] Romero P, Obradovic Z, Li X, Garner EC, Brown CJ, Dunker AK (2001). Sequence complexity of disordered protein. Proteins.

[B63] Vucetic S, Brown CJ, Dunker AK, Obradovic Z (2003). Flavors of protein disorder. Proteins.

[B64] Obradovic Z, Peng K, Vucetic S, Radivojac P, Brown CJ, Dunker AK (2003). Predicting intrinsic disorder from amino acid sequence. Proteins.

[B65] Goh GK-M, Dunker AK, Uversky VN (2008). Protein intrinsic disorder toolbox for comparative analysis of viral proteins. BMC Genomics.

[B66] Reid A (2005). The effects of the 1918–1919 influenza pandemic on infant and child health in Derbyshire. Med Hist.

[B67] Russell RJ, Gamblin SJ, Haire LF, Stevens DJ, Xiao B, Ha Y, Skehel JJ (2004). H1 and H7 influenza haemagglutinin structures extend a structural classification of haemagglutinin subtypes. Virology.

[B68] Di Trani LBB, Cordioli P, Muscillo M, Vignolo E, Moreno A, Tollis M (2004). Molecular characterization of low pathogenicity H7N3 avian influenza viruses isolated in Italy. Avian Dis.

[B69] Wan H, Sorrell E, Song H, Hossain M, Ramirez-Nieto G, Monne I, Stevens J, Cattoli G, Capua I, Chen L (2008). Replication and transmission of H9N2 influenza viruses in ferrets: evaluation of pandemic potential. PLoS ONE.

[B70] Matrosovich MN, Krauss S, Webster RG (2001). H9N2 influenza A viruses from poultry in Asia have human virus-like receptor specificity. Virology.

[B71] Hatta M, Gao P, Halfmann P, Kawaoka Y (2001). Molecular basis for high virulence of Hong Kong H5N1 influenza A viruses. Science.

[B72] Guan Y, Poon LL, Cheung CY, Ellis TM, Lim W, Lipatov AS, Chan KH, Sturm-Ramirez KM, Cheung CL, Leung YH (2004). H5N1 influenza: a protean pandemic threat. Proc Natl Acad Sci USA.

[B73] Kaverin NV, Rudneva IA, Ilyushina NA, Lipatov AS, Krauss S, Webster RG (2004). Structural differences among hemagglutinins of influenza A virus subtypes are reflected in their antigenic architecture: analysis of H9 escape mutants. J Virol.

[B74] Kaverin NV, Rudneva IA, Ilyushina NA, Varich NL, Lipatov AS, Smirnov YA, Govorkova EA, Gitelman AK, Lvov DK, Webster RG (2002). Structure of antigenic sites on the haemagglutinin molecule of H5 avian influenza virus and phenotypic variation of escape mutants. J Gen Virol.

[B75] Rudneva I, Ilyushina N, Timofeeva T, Webster R, Kaverin N (2005). Restoration of virulence of escape mutants of H5 and H9 influenza viruses by their readaptation to mice. J Gen Virol.

[B76] Goh GKM, Uversky V, Dunker AK (2008). A Comparative Analysis of Viral Matrix Proteins Using Disorder Predictor. Virol J.

[B77] Goh GKM, Dunker AK, Uversky V (2008). Protein Intrinsic Disorder Toolbox for Comparative Analysis of Viral Proteins. BMC Genomics.

[B78] Yuen KY, Wong SS (2005). Human infection by avian influenza A H5N1. Hong Kong Med J.

[B79] Yuen KY, Chan PK, Peiris M, Tsang DN, Que TL, Shortridge KF, Cheung PT, To WK, Ho ET, Sung R, Cheng AF (1998). Clinical features and rapid viral diagnosis of human disease associated with avian influenza A H5N1 virus. Lancet.

[B80] Chan PK (2002). Outbreak of avian influenza A(H5N1) virus infection in Hong Kong in 1997. Clin Infect Dis.

[B81] Us D (2008). [Cytokine storm in avian influenza]. Mikrobiyol Bul.

[B82] Ha Y, Stevens DJ, Skehel JJ, Wiley DC (2001). X-ray structures of H5 avian and H9 swine influenza virus hemagglutinins bound to avian and human receptor analogs. Proc Natl Acad Sci USA.

[B83] Reid AH, Taubenberger JK, Fanning TG (2004). Evidence of an absence: the genetic origins of the 1918 pandemic influenza virus. Nat Rev Microbiol.

[B84] Webster RG, Sharp GB, Claas EC (1995). Interspecies transmission of influenza viruses. Am J Respir Crit Care Med 1995.

[B85] Gamblin SJ, Haire LF, Russell RJ, Stevens DJ, Xiao B, Ha Y, Vasisht N, Steinhauer DA, Daniels RS, Elliot A (2004). The structure and receptor binding properties of the 1918 influenza hemagglutinin. Science.

[B86] Lin YP, Shaw M, Gregory V, Cameron K, Lim W, Klimov A, Subbarao K, Guan Y, Krauss S, Shortridge K (2000). Avian-to-human transmission of H9N2 subtype influenza A viruses: relationship between H9N2 and H5N1 human isolates. Proc Natl Acad Sci USA.

[B87] Loo YM, Gale M (2007). Influenza: Fatal immunity and the 1918 virus. Nature.

[B88] Berman HM, Westbrook Z, Feng G, Bhat H, Weissig H, Sindyalov LN, Bourne PE (2000). The Protein Data Bank. Nucleic Acids Res.

[B89] Herráez A (2006). Biomolecules in the computer: Jmol to the rescue. Biochemistry and Molecular Biology Education.

